# Network pharmacology to unveil the mechanism of suanzaoren decoction in the treatment of alzheimer’s with diabetes

**DOI:** 10.1186/s41065-023-00301-z

**Published:** 2024-01-03

**Authors:** Tao Chen, Yining Lei, Manqin Li, Xinran Liu, Lu Zhang, Fei Cai, Xiaoming Gong, Ruyi Zhang

**Affiliations:** 1 Department of Pharmacy, Xianning Central Hospital, The First Affiliate Hospital of Hubei University of Science and Technology, Xianning, 437100 Hubei China; 2 Hubei Key Laboratory of Diabetes and Angiopathy, Hubei University of Science and Technology, Xianning, 437100 China; 3 School of Basic Medical Sciences, Xianning Medical College, Hubei University of Science and Technology, Xianning, 437100 China

**Keywords:** Network pharmacology, Molecular dynamics simulation, Alzheimer’s with diabetes, Licochalcone A, Isorhamnetin, Kaempferol

## Abstract

**Background:**

Suanzaoren Decoction (SZRD), a well-known formula from traditional Chinese medicine, has been shown to have reasonable cognitive effects while relaxing and alleviating insomnia. Several studies have demonstrated significant therapeutic effects of SZRD on diabetes and Alzheimer’s disease (AD). However, the active ingredients and probable processes of SZRD in treating Alzheimer’s with diabetes are unknown. This study aims to preliminarily elucidate the potential mechanisms and potential active ingredients of SZRD in the treatment of Alzheimer’s with diabetes.

**Methods:**

The main components and corresponding protein targets of SZRD were searched on the TCMSP database. Differential gene expression analysis for diabetes and Alzheimer’s disease was conducted using the Gene Expression Omnibus database, with supplementation from OMIM and genecards databases for differentially expressed genes. The drug-compound-target-disease network was constructed using Cytoscape 3.8.0. Disease and SZRD targets were imported into the STRING database to construct a protein-protein interaction network. Further, Gene Ontology and Kyoto Encyclopedia of Genes and Genomes analyses were performed on the intersection of genes. Molecular docking and molecular dynamics simulations were conducted on the Hub gene and active compounds. Gene Set Enrichment Analysis was performed to further analyze key genes.

**Results:**

Through the Gene Expression Omnibus database, we obtained 1977 diabetes related genes and 622 AD related genes. Among drugs, diabetes and AD, 97 genes were identified. The drug-compound-target-disease network revealed that quercetin, kaempferol, licochalcone a, isorhamnetin, formononetin, and naringenin may be the core components exerting effects. PPI network analysis identified hub genes such as *IL6, TNF, IL1B, CXCL8, IL10, CCL2, ICAM1, STAT3*, and *IL4*. Gene Ontology and Kyoto Encyclopedia of Genes and Genomes analyses showed that SZRD in the treatment of Alzheimer’s with diabetes is mainly involved in biological processes such as response to drug, aging, response to xenobiotic, and enzyme binding; as well as signaling pathways such as Pathways in cancer, Chemical carcinogenesis - receptor activation, and Fluid shear stress and atherosclerosis. Molecular docking results showed that licochalcone a, isorhamnetin, kaempferol, quercetin, and formononetin have high affinity with *CXCL8, IL1B*, and *CCL2*. Molecular dynamics simulations also confirmed a strong interaction between *CXCL8* and licochalcone a, isorhamnetin, and kaempferol. Gene Set Enrichment Analysis revealed that *CXCL8, IL1B*, and *CCL2* have significant potential in diabetes.

**Conclusion:**

This study provides, for the first time, insights into the active ingredients and potential molecular mechanisms of SZRD in the treatment of Alzheimer’s with diabetes, laying a theoretical foundation for future basic research.

**Graphical Abstract:**

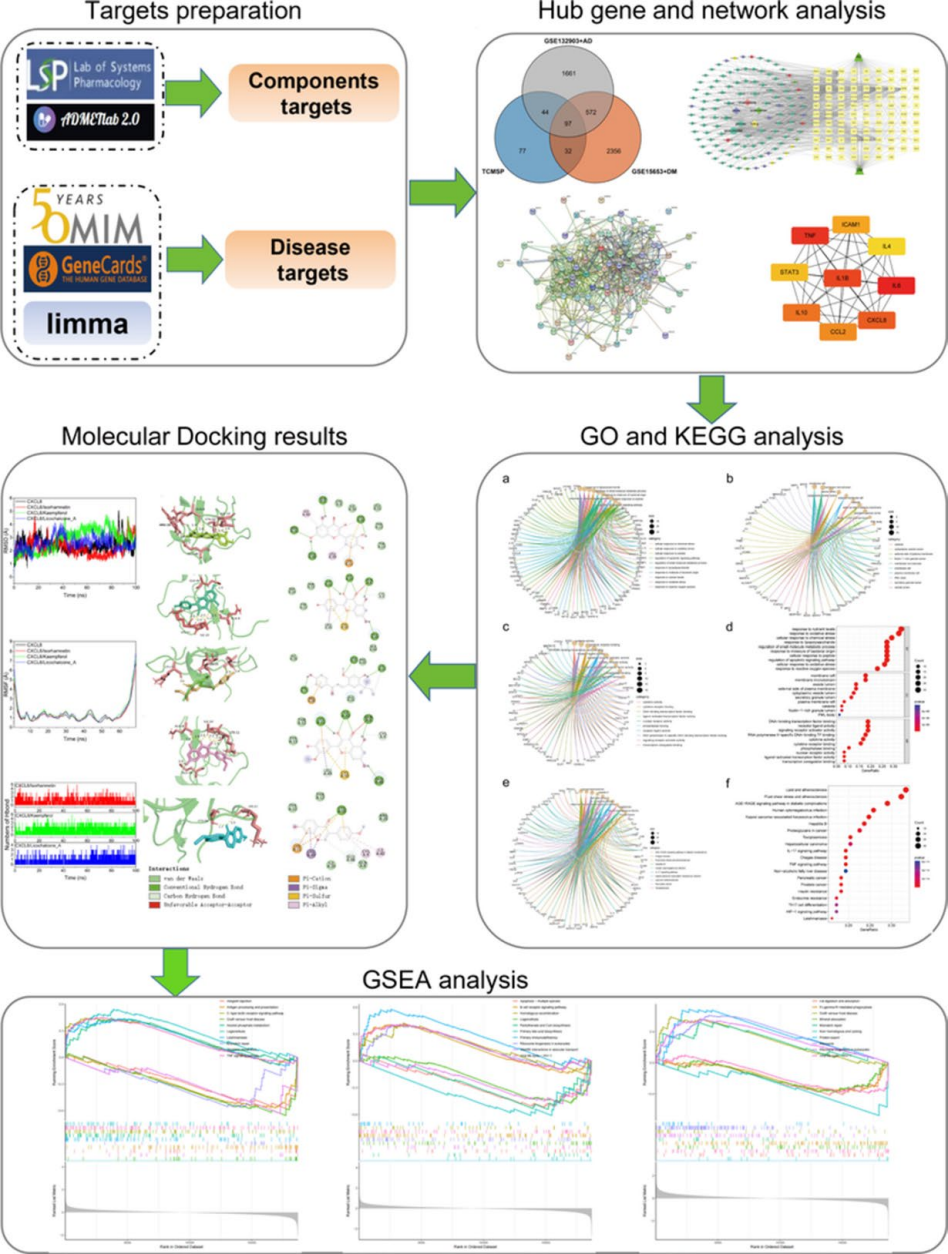

## Highlights

• SZRD may improve Alzheimer’s with diabetes through potential active ingredients and hub genes.

• licochalcone a, isorhamnetin, kaempferol, quercetin, and formononetin are potential active ingredients of SZRD for the treatment of Alzheimer’s with diabetes.

*• IL6, TNF, IL1B, CXCL8, IL10, CCL2, ICAM1, STAT3* and *IL4* are hub genes and have a strong binding capacity to potential active ingredients.

## Introduction

Diabetes is increasingly prevalent globally, with the highest incidence observed in the age group of 75–79 years old [1]. In 2021, global healthcare expenditures associated with diabetes were estimated at $966 billion, with a projected increase to $10.54 trillion by 2045 [2]. AD is the leading neurodegenerative cause of dementia, responsible for 50–70% of cases of neurodegenerative dementia. The estimated global prevalence of dementia is around 44 million people, and this number is projected to triple by 2050 [3]. Diabetes can result in brain tissue damage and cognitive dysfunction, and it is also recognized as a risk factor for AD [4, 5]. Epidemiological studies have confirmed that individuals with diabetes have an elevated risk of dementia compared to those without diabetes [6]. Studies have shown that the risk of dementia in patients with diabetes is associated with the prevalence of mild cognitive dysfunction progressing to dementia [7]. Cognitive dysfunction in individuals with diabetes plays a significant role in the relationship between diabetes and AD. Hence, the discovery of novel therapeutic drugs for managing Alzheimer’s with diabetes holds profound implications for individuals with both diabetes and AD.

Traditional Chinese medicine has garnered growing attention as a potential treatment for cognitive impairments associated with diabetes. SZRD is a Chinese herbal formula that originated from the book “Jin Kui Yao Lue” written by Zhang Zhongjing in the Han Dynasty, and it comprises five medicinal herbs, namely Ziziphus jujuba Mill (Suanzaoren, SZR), Glycyrrhiza uralensis Fisch (Gancao, GC), Anemarrhena asphodeloides Bunge (Zhimu, ZM), Poria cocos (Schw.) Wolf. (Fuling, FL), and Ligusticum acuminatum Franch. (Chuanxiong, CX), known for their calming, nourishing, and insomnia-treating effects. Despite being a traditional herbal prescription for insomnia, SZRD has also been utilized in the treatment of other ailments. Studies have demonstrated that SZRD can elevate the levels of neurotransmitters, including 5-HT, DA, and GABA, in the brain, potentially contributing to its effectiveness in treating insomnia [8]. Moreover, SZRD has exhibited the ability to enhance learning and memory in mouse models of AD [9]. In addition, Glycyrrhiza uralensis Fisch [10] and Anemarrhena asphodeloides Bunge [11] have been found to improve diabetes. We hypothesize that SZRD holds promising potential for the treatment of cognitive impairments and diabetes. Further investigation and elucidation of its underlying compounds and targets are warranted to comprehensively understand its therapeutic effects in cognitive disorders.

The network pharmacology approach was originally introduced as a novel avenue for identifying new drug candidates and repurposing existing ones from intricate network models [12]. The study of herbal medicines in disease treatment poses unique challenges due to their complex composition, multiple targets, and broad therapeutic signaling pathways [13]. Hence, utilizing network pharmacology to elucidate the effects of traditional herbal formulations on complex diseases and predict potential compounds and targets represents a crucial approach. Molecular docking, as a structure-based and computer-assisted drug design method, holds a pivotal role in drug discovery and research [14]. Molecular dynamics simulation, capable of capturing the real-time trajectory of macromolecules [15], has found widespread use in various fields, including biology. We have employed a multi-faceted approach encompassing bioinformatics, network pharmacology, molecular docking, and molecular dynamics simulation to precisely target herbal medicines for disease treatment.

**Fig. 1:**
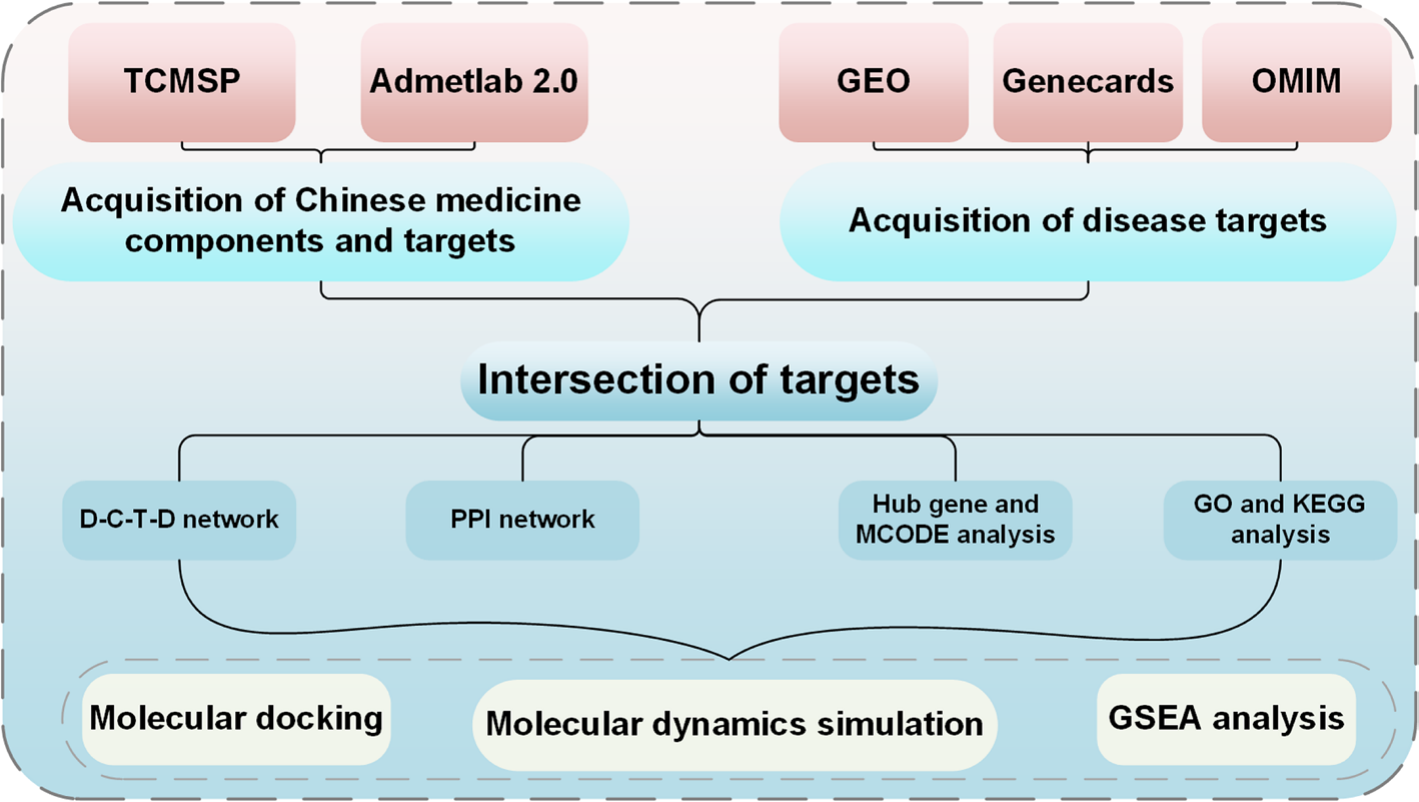
Workflow of the network pharmacological investigation strategy of SZRD in the treatment of diabetes and AD. Four parts include target preparation, Hub gene, and network analysis, GO and KEGG analysis, molecular docking verification, and GSEA analysis

SZRD has demonstrated promising efficacy in improving cognitive dysfunction, and some studies have also reported its potential in lowering blood glucose levels. We posit that SZRD may hold promise as an herbal medicine for treating Alzheimer’s with diabetes, albeit challenges persist in identifying its composition and targets. Therefore, this study has integrated bioinformatics, network pharmacology, and molecular docking to predict the active compounds, potential targets, and underlying molecular mechanisms of SZRD in Alzheimer’s with diabetes, aiming to contribute to the field of traditional Chinese medicine and provide a reference and foundation for future researchers. The experimental workflow is illustrated in Fig. 1.

## Methods

### Component target prediction

Traditional Chinese Medicine Pharmacology Systematic Pharmacology (TCMSP, https://old.tcmsp-e.com/tcmsp.php) database based on the systematic pharmacology of Chinese medicine [16], It contains a rich variety of herbs, chemical components, and 12 important properties related to ADME required for drug screening and evaluation. Oral bioavailability (OB) is the rate and extent to which a drug is absorbed into the circulation. Drug similarity (DL) is the similarity between the component and the marketed drug [17]. Based on the OB principle, we used OB ≥ 30% and DL ≥ 0.18 in the TCMSP component screening [18], while reviewing the literature for further collection. Uniport (http://www.uniprot.org/) was used to transform the obtained targets in terms of gene symbols. Admetlab 2.0 (https://admetmesh.scbdd.com/) is an online pharmacokinetic and toxicity prediction program [19]. It was used to predict drug absorption, distribution, metabolism, excretion, and toxicity profiles. The SMILES of the compounds are entered into Admetlab 2.0 to obtain the absorption, distribution, and toxicity profiles of the components, etc.

### Disease target prediction

The Gene Expression Omnibus Dataset (GEO, http://www.ncbi.nlm.nih.gov/geo/) database is a website for disease gene expression, and we used “diabetes” and " Alzheimer’s disease " as search strategies, limiting species to “homo spines”. We selected datasets for diabetes (GSE15653) and AD (GSE132903), using “limma”, “pheatmap”, “ggplot2 “, “ggsci”, “dplyr”, “org.Hs.eg.db” and “patchwork” packages were used to visualize the differentially expressed genes(DEG), volcanoes and heatmaps, and the threshold values for DEG identification were |logfc|>0.5 and *P* < 0.05 [20]. The search for AD and diabetes-related targets was performed by Genecards (https://www.genecards.org/) and OMIM (https://www.omim.org/) using the keywords “AD” and “diabetes”. The Relevance score was restricted to the second average in Genecards to accurately screen the relevant targets, and all the targets of diabetes and AD were combined and removed the duplicate values to draw a Venn diagram.

### Construction of drug-compound-target-disease (D-C-T-D) networks and protein-protein interaction (PPI) networks

To better analyze the connection between drugs, components, and targets, we built a “D-C-T-D” network in Cytoscape 3.8.0 software, Cytoscape can calculate the parameters of each node in the network graph, such as degree, betweenness centrality (BC), closeness centrality (CC), etc. [21, 22]. All these parameters allow an in-depth analysis of the properties of the nodes in the interaction network, and we used the degree, BC, and CC to filter the top-ranked components and targets, regarded as playing a central role. The STRING network was constructed (https://string-db.org/) as a database for analyzing the relationships between proteins [23]. Using this database, we construct a “PPI” network that captures the interactions between intersecting targets and collects targets with strong connections. The scoring condition was set to > 0.70 and the selected target proteins were restricted to Homo sapiens. In the PPI network, edges represent protein-protein associations, and the more lines there are, the greater the correlation. Screening of key targets and analysis followed.

### Hub gene extraction and MCODE analysis

PPI networks consist of nodes, edges, and connecting lines, and it is generally believed that the most critical nodes are hub genes. Cytohubba (http://apps.cytoscape.org/apps/cytohubba) is a new Cytoscape plugin for ranking and extracting biological networks based on various network features for central or potential target elements based on various network features. Cytohubba has 11 methods to study networks from different perspectives, of which maximum population centrality (MCC) is the best one [24]. We used the MCC of Cytohubba to identify the top 10 hub genes from PPI networks. Metascape (https://metascape.org/gp/index.html) is an efficient tool in the era of big data, combining functional enrichment, interactome analysis, gene annotation, and member search to provide a comprehensive gene list annotation and analysis resource [25]. We entered all intersecting genes into Metascape for MCODE analysis.

### GO and KEGG pathway enrichment analysis

R packages such as “org.Hs.eg.db”, “clusterprofiler”, “enrichplot”, “ggplot2”, “ggnewscale” and “DOSE” were used for GO and KEGG enrichment analysis and result plotting. GO is divided into three categories, namely biological process (BP), Cellular component (CC), and Molecular function (MF), and KEGG enrichment analysis is a way to analyze the pathway enrichment of genes. All intersecting targets were analyzed and the top ten biological processes of BP, CC, MF and KEGG in GO were selected for graphical visualization.

### Molecular docking

Molecular docking is one of the most commonly used methods for structure-based drug design [26]. Molecular docking of active compounds and hub genes was performed using autodocktools-1.5.7 software. First, the 3D structure of the active compound was downloaded from PubChem (http://Pubchem.ncbi.nlm.nih.gov/); then, the water was removed, and hydrogen atoms were added and converted to PDBQT format using autodocktools. Download the PDB format of the relevant target at RSCB-PDB (https://www.rcsb.org/) and remove the ligands and water molecules using PYMOL software. Then, import them into autodocktools, add hydrogen atoms, calculate the charges, convert them to PDBQT format and perform molecular flexible docking, selecting the docking model with the lowest binding energy among them. Finally, the 3D docking results were visualized using PYMOL software and the 2D docking results were visualized using Discovery Studio software 2017. Among them, a docking score AFFINITY <-4.25 KCAL/MOL^−1^ considered a binding activity between ligand and target, a score <-5.0 KCAL/MOL^−1^ indicated a better binding activity, and a score <-7.0 KCAL/MOL^−1^ a strong docking activity between the two [27].

### Molecular dynamics simulation

All-atom molecular dynamics simulations were performed separately based on the small molecule and protein complexes obtained from the above docking as initial structures, and the simulations were performed using AMBER 18 software [28]. Before the simulations, the charges of the small molecules were obtained by the antechamber module and Hartree-Fock (HF) SCF/6-31G* calculations of the Gaussian 09 software [29, 30]. The small molecule and protein force fields were used for GAFF2 small molecule force field and ff14sb protein force field, respectively [31, 32]. The leap module was used for each system to add hydrogen atoms to the system, a truncated octahedral TIP3P solvent cartridge was added at a distance of 10 Å from the system [33], and Na+/Cl- was added to balance the system charge. A 200 ps ramp-up of the system was performed to slowly increase the system temperature from 0 to 298.15 K. A 500 ps simulation of the NVT (isothermal isomer) tether was performed with the system maintained at 298.15 K. In the case of NPT (isothermal isobaric), equilibrium simulations were performed for the whole system for 500 ps. Finally, 100 ns of NPT (isothermal isobaric) tethering simulations were performed separately. For the simulations, the non-bond truncation distance is set to 10 Å. The Particle mesh Ewald (PME) method is used to calculate the long-range electrostatic interaction [34], the SHAKE method is used to limit the bond length of hydrogen atoms [35], and the Langevin algorithm is used for temperature control [36], where the collision frequency γ is set to 2 ps^-^[1]. The system pressure is 1 atm, the integration step is 2 fs, and the trajectories are saved at 10 ps intervals for subsequent analysis. The traces were saved at 10 ps intervals for subsequent analysis.

The binding free energies between proteins and ligands for all systems were calculated by the MM/GBSA method [37–40]. The long-time molecular dynamics simulations may not be conducive to the accuracy of MM/GBSA calculations [38]. Therefore, the MD trajectory of 90–100 ns was used as the calculation in this study with the following equations.

1 $${\Delta\mathrm{G}}_{\mathrm{bind}}=\;{\Delta\mathrm{G}}_{\mathrm{complex}}\;-\;{({\Delta\mathrm{G}}_{\mathrm{receptor}}\;+\;{\Delta\mathrm{G}}_{\mathrm{ligand}})}\;=\;{\Delta\mathrm{E}}_{\mathrm{internal}}\;+\;{\Delta\mathrm{E}}_{\mathrm{VDW}}\;+\;{\Delta\mathrm{E}}_{\mathrm{elec}}\;+\;{\Delta\mathrm{G}}_{\mathrm{GB}}\;+\;{\Delta\mathrm{G}}_{\mathrm{SA}}$$

In Eq. (1), ${{\Delta }\text{E}}_{\text{i}\text{n}\text{t}\text{e}\text{r}\text{n}\text{a}\text{l}}$ denotes internal energy、${{\Delta }\text{E}}_{\text{V}\text{D}\text{W}}$denotes van der Waals interaction, and ${{\Delta }\text{E}}_{\text{e}\text{l}\text{e}\text{c}}$ denotes electrostatic interaction. The internal energy includes E_bond_, E_angle_, and E_torsion_;${{\Delta }\text{G}}_{\text{G}\text{B}}$ and ${{\Delta }\text{G}}_{\text{G}\text{A}}$ are collectively referred to as the solventization free energy. Among them, G_GB_ is the polar solvation free energy and G_SA_ is the non-polar solvation free energy. For ${{\Delta }\text{G}}_{\text{G}\text{B}}$, the GB model (igb = 2) developed by Nguyen [41] is used for the calculation. The nonpolar solvation free energy (Δ*G*_*SA*_) is calculated based on the product of surface tension (γ) and solvent accessible surface area (SA), Δ*G*_*SA*_= 0.0072 × ΔSASA [42]. The entropy change is neglected in this study due to high computational resources and low precision; this study was neglected [37, 38].

### Gene Set Enrichment Analysis (GSEA)

The Gene Set Enrichment Analysis [43] was performed by “ggplot2”, “limma”, “ggsci”, “org.Hs.eg.db” and “patchwork”. We selected the top 3 genes of molecular docking results for GSEA single gene analysis, and the disease group of GSE15932 (diabetes) was selected for the signature gene set. Enrichment scores (ES) were calculated based on weighted Kolmogorov-Smirnov class statistics, and their magnitude reflects the correlation between gene set and phenotype. A higher ES of a gene set implies a higher likelihood that the gene set is enriched in a specific phenotype [44].

## Result

### Data collection of disease and drug components

**Fig. 2:**
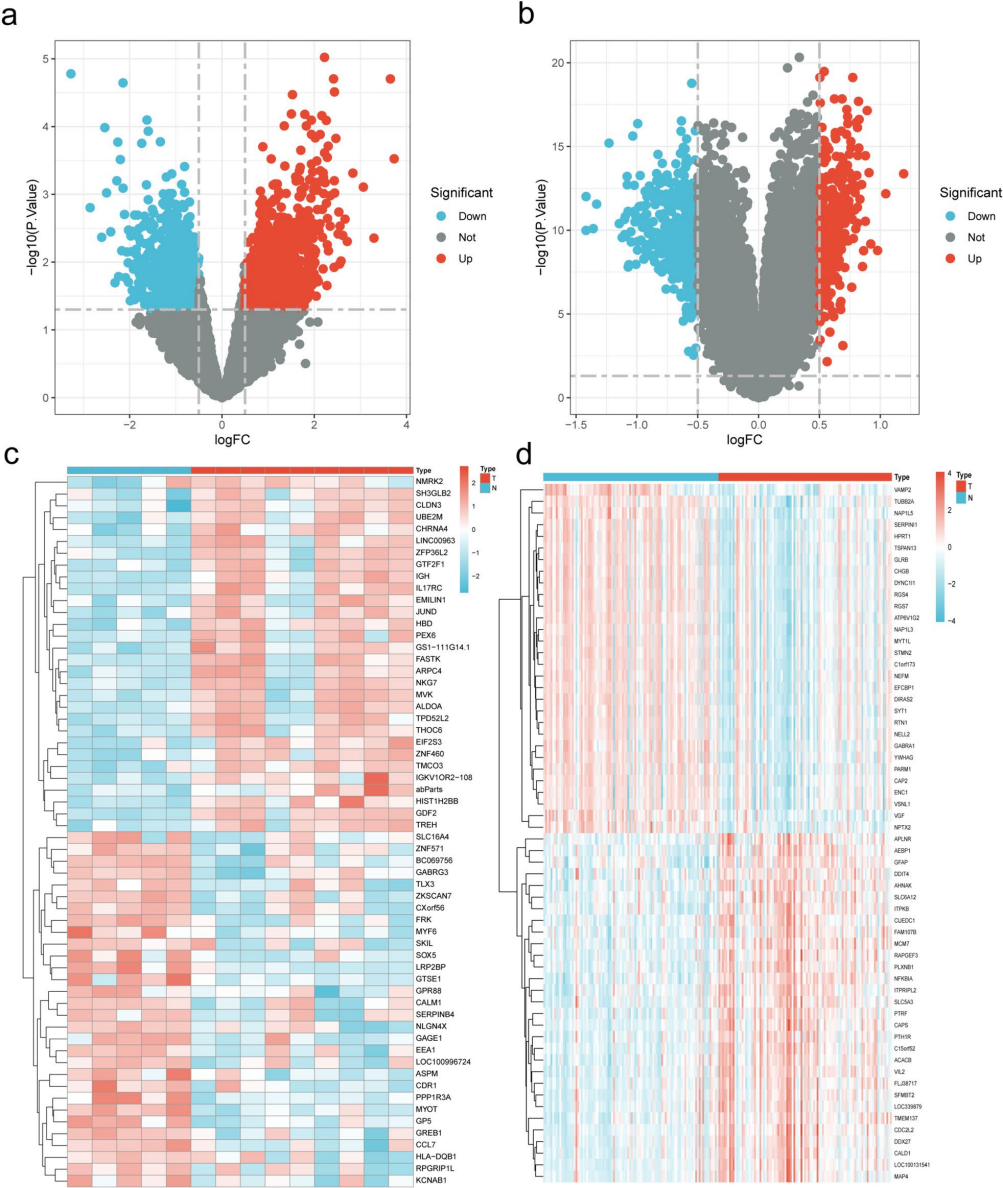
Screening of diabetes and AD targets. Differential volcano plot showing the gene distribution of disease samples, GSE15653 (**a**) and GSE132903(**b**), The heat map shows the top 30 up- and down-regulated DEGs, GSE15653 (**c**) and GSE132903 (**d**)

First, to precisely identify the targets of diabetes and AD. We retrieved DEGs by comparing the differential gene expression levels between control and disease groups. GSE15653 selected 5 control and 9 diabetes group samples and analyzed 995 up-regulated and 982 down-regulated genes (Fig. 2a); GSE132903 selected 98 control and 97 AD group samples and analyzed 336 down-regulated genes and 286 up-regulated genes (Fig. 2c). The DEGs of the top 30 up and down-regulated genes (Fig. 2b, d) were also shown with heat maps. 1051 diabetes targets and 1354 AD targets were identified in genecards; 225 diabetes targets and 546 AD targets were identified in OMIM as complementary targets for diabetes and AD.

**Fig. 3:**
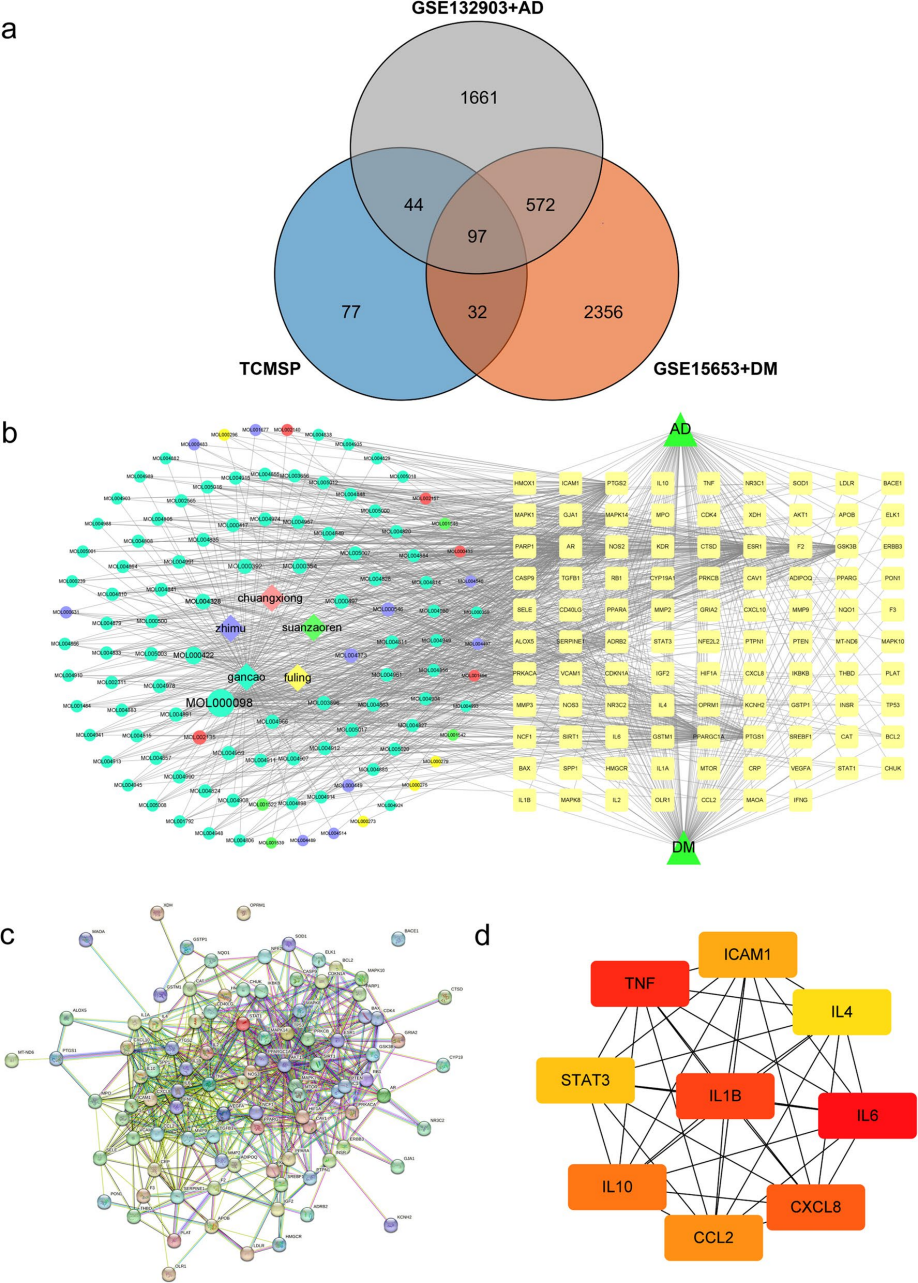
Network pharmacology. **a** Venn diagram of 97 intersecting targets between drug, diabetes, and AD, (**b**) D-C-T-D network diagram, gancao (blue green), zhimu (blue-violet), chuangxiong (red), suanzaoren (green), fuling (yellow), and targets (light yellow). **c** PPI network of 97 intersecting targets. **d** The top 9 gene is selected MCC from the PPI network

To identify the potentially active chemical components in Chinese medicine. The chemical composition of each herbal drug collected from TCMSP was initially screened using the OB and DL properties of the drugs. The literature was also reviewed and a total of 108 potential components were identified. Among them, SZR and FL both had 4 components, ZM had 11 components, CX had 6 components and GC had 85 components, MOL000359 was common to GC and CX, and MOL000422 was common to ZM and GC. After the uniport transformation of the targets, a total of 253 targets corresponding to the compounds were found in TCMSP. The intersection of 97 targets among drug, diabetes, and AD were obtained by making a Venn diagram (Fig. 3a).

### Construction of D-C-T-D networks and PPI networks

These intersection targets and corresponding components were imported into Cytoscape to draw a D-C-T-D network graph, including 212 nodes and 1114 edges (Fig. 3b). Network analysis allows the identification of potentially active compounds for drug therapeutic action. The network was analyzed by Cytoscape, and MOL000098 (degree: 69), MOL000422 (degree: 26), MOL004328 (degree: 19), MOL000392 (degree: 16), MOL000354 (degree: 16), and MOL000497 (degree: 15) were the key compounds (Table 1). The 97 intersecting targets were also imported into the string database to obtain the PPI network graph, with 97 points and 641 edges (Fig. 3c). These results suggest that SZRD may act on Alzheimer’s with diabetes through these compounds, and the existence of a strong association between these targets suggests that SZRD can have a therapeutic effect on Alzheimer’s with diabetes through the ability of multiple compounds and multiple targets thus.

**Table 1:**
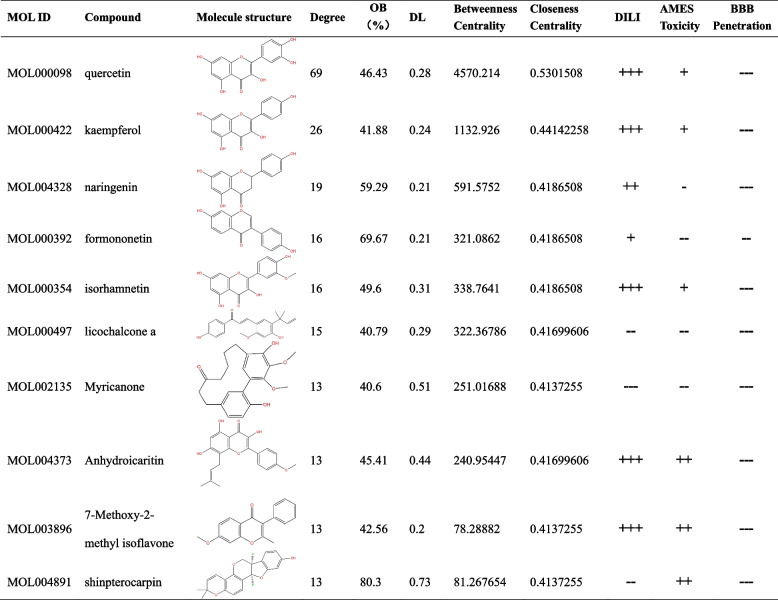
Top ten compounds information of D-C-T-D network

The prediction probability values are transformed into six symbols: 0–0.1(---), 0.1–0.3(--), 0.3–0.5(-), 0.5–0.7(+), 0.7–0.9(++), and 0.9–1.0(+++). The token ‘+++’ or ‘++’ represents the molecule is more likely to be toxic or defective, while ‘**---**’ or ‘**--**’ represents nontoxic or appropriate. *OB *Oral Bioavailability, *DL *Drug-Likeness, *DILI *Drug induced liver injury, *AMES *The bacterial reverse mutation test

### Hub gene extraction and MCODE analysis

**Fig. 4:**
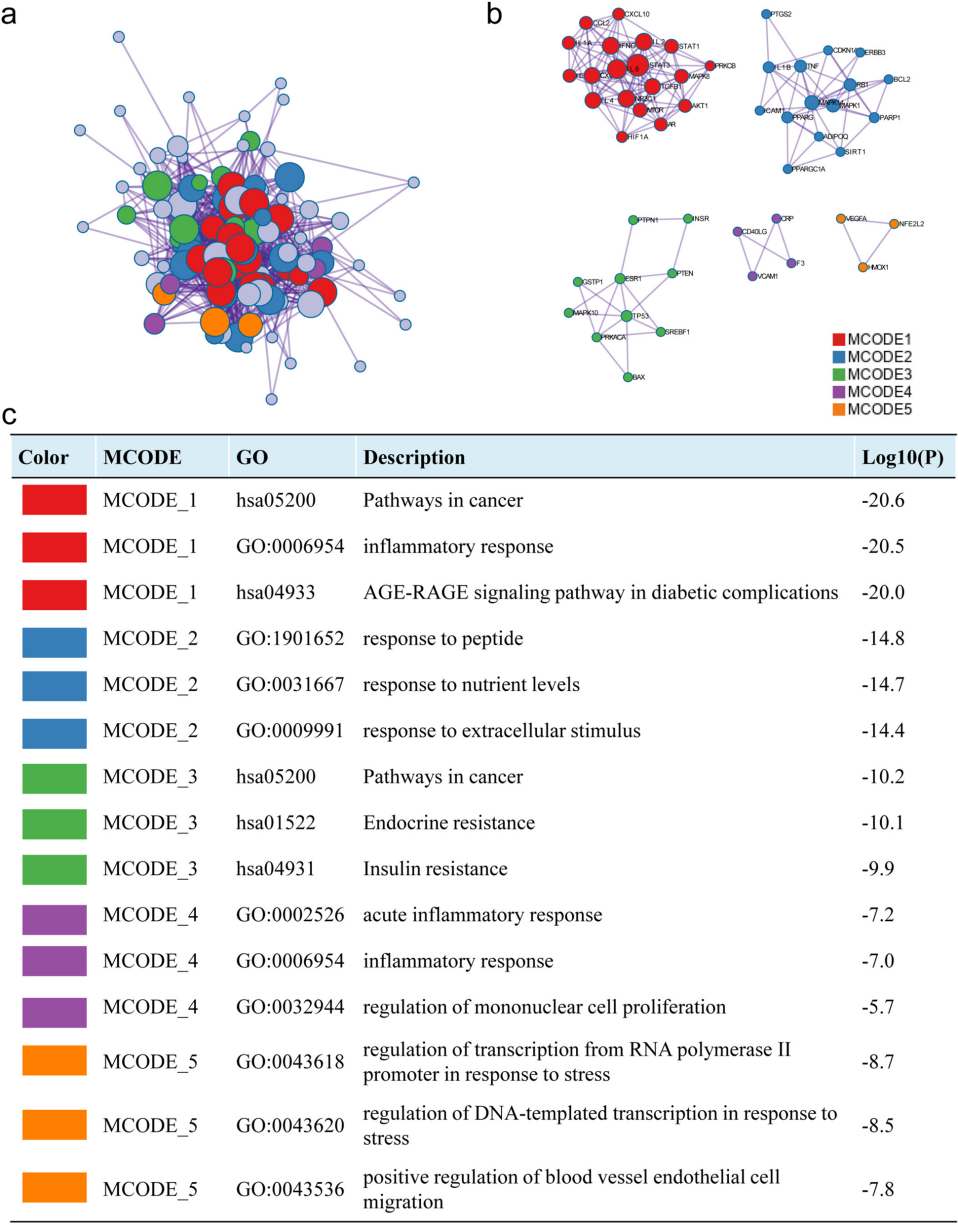
Results of MCODE analysis. **a** Network diagram of PPI after MCODE clustering analysis. **b** Network diagram of MCODE clustering separately. **c** The entries, categories, and corresponding log p-values of each cluster were obtained from the MCODE cluster analysis

Most of the interconnected nodes in a PPI network are considered to be hub genes in the PPI network. According to the PPI network analysis of the Cytohubba plugin in Cytoscape, we listed the top 9 (9.28%) DEGs as the most influential genes (Fig. 3d). The hub gene is namely *IL6* (Score: 9.75E + 08), *TNF* (Score: 9.75E + 08), *IL1B* (Score: 9.74E + 08), *CXCL8* (Score: 9.74E + 08), *IL10* (Score: 9.74E + 08), *CCL2* (Score: 9.74E + 08), *ICAM1* (Score: 9.74E + 08), *STAT3* (Score: 9.66E + 08) and *IL4* (Score: 9.62E + 08). These hub genes may be potential biomarkers, which may also be new disease treatment strategies. To better understand these targets, we performed MCODE module analysis with the help of Metascape to gain more insight into the degree of target clustering (Fig. 4). The results of MCODE clustering analysis showed that these targets were mainly clustered in pathway in cancer, inflammatory response, AGE-RAGE signaling pathway in diabetic complications, inflammatory response, and response to peptide.

### GO and KEGG enrichment analysis

**Fig. 5:**
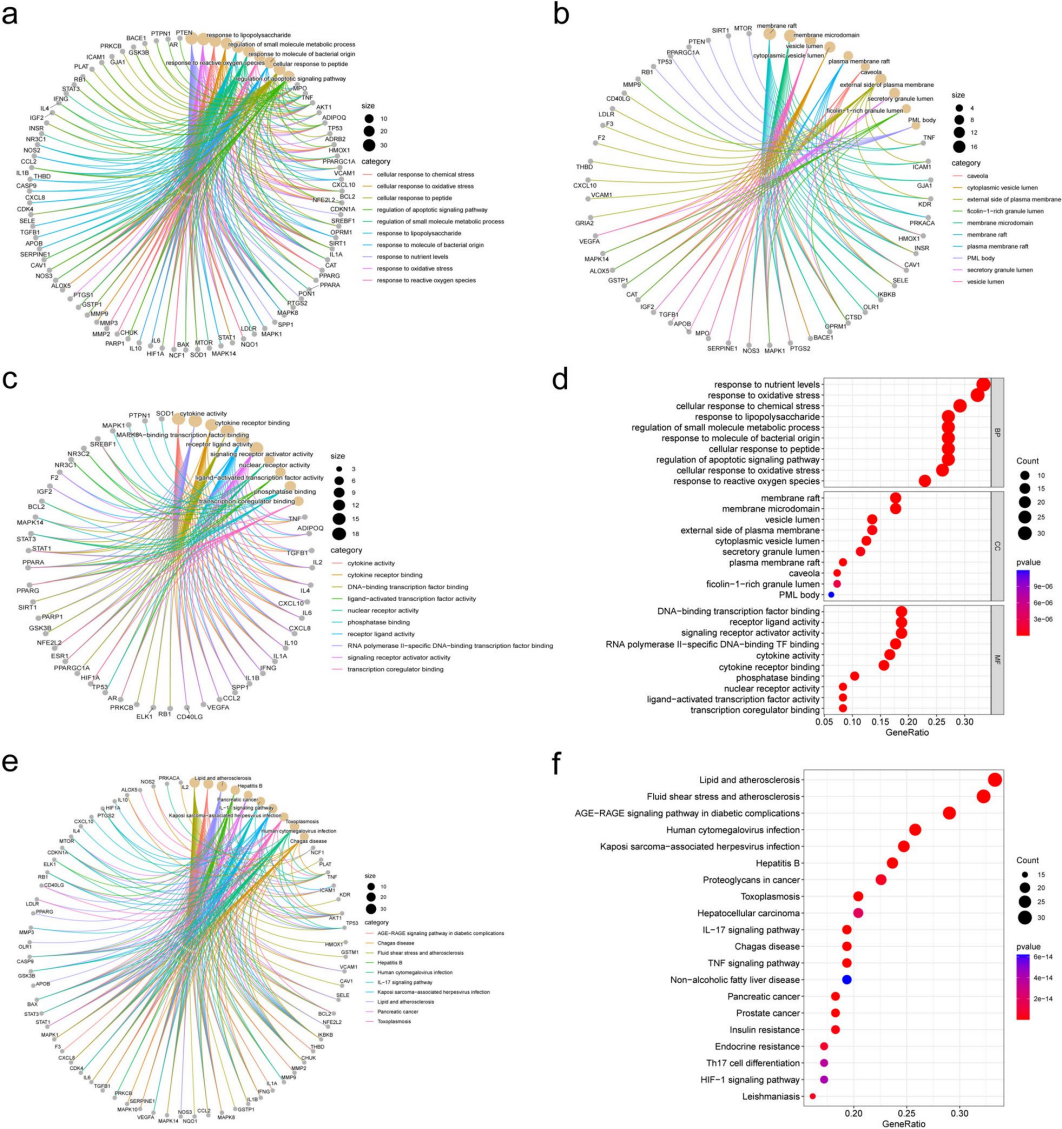
GO and KEGG enrichment analysis results. network plots between the top 10 pathways for each category in GO enrichment analysis BP (**a**), CC (**b**), and MF (**c**). **d** Bubble plots of GO enrichment analysis. **e** Network diagram of the top 10 pathways analyzed by KEGG enrichment. **f** Bubble plots of KEGG enrichment analysis

R was used to perform GO and KEGG enrichment analysis to identify intersecting genes that share the biological significance and enrichment pathways highlighted in this study. Gene ontology considers gene functions and their components, providing a broad resource of computable knowledge. Gene ontology analysis was performed for three categories (biological processes, cellular components, and molecular functions) and the GO database was selected as the annotation source. KEGG analysis reveals the response of an organism to its intrinsic modifications. It is a modeling technique to demonstrate the interactions between various diseases through underlying molecular or biological processes. Table 2 summarizes the top 10 terms in the categories of biological processes, molecular functions, and cellular components; Table 3 summarizes the top 20 terms in the KEGG pathway. We plotted network diagrams (Fig. 5a, b, c, e) and bubble diagrams (Fig. 5d, f) for the top ten GO entries and KEGG pathways and corresponding targets. These results can reveal that SZRD can function in multiple biological processes and signaling pathways, probably mainly enriched in biological processes such as response to nutrient levels, response to oxidative stress, and cellular response to chemical stress. KEGG enrichment analysis showed that the KEGG enrichment was mainly in multiple pathways such as Fluid shear stress and atherosclerosis, AGE-RAGE signaling pathway in diabetic complications, and Lipid and atherosclerosis.

**Table 2:** GO analysis; divided into three categories (the first 10 articles with the smallest *p*-value)

GO ID	Term	count	*P*-values	Category
GO:0031667	response to nutrient levels	32	1.43E-27	Biological process
GO:0006979	response to oxidative stress	31	1.99E-27	Biological process
GO:0062197	cellular response to chemical stress	28	2.95E-26	Biological process
GO:0034599	cellular response to oxidative stress	25	3.74E-24	Biological process
GO:0032496	response to lipopolysaccharide	26	9.96E-24	Biological process
GO:0062012	regulation of small molecule metabolic process	26	9.96E-24	Biological process
GO:0000302	response to reactive oxygen species	22	2.25E-23	Biological process
GO:0002237	response to molecule of bacterial origin	26	4.67E-23	Biological process
GO:1901653	cellular response to peptide	26	1.16E-22	Biological process
GO:0045121	membrane raft	26	1.24E-22	Biological process
GO:0098857	membrane microdomain	17	2.88E-13	Cellular component
GO:0031983	vesicle lumen	17	3.03E-13	Cellular component
GO:0060205	cytoplasmic vesicle lumen	13	6.07E-09	Cellular component
GO:0044853	plasma membrane raft	12	5.48E-08	Cellular component
GO:0005901	Caveola	8	7.45E-08	Cellular component
GO:0009897	external side of the plasma membrane	7	1.39E-07	Cellular component
GO:0034774	secretory granule lumen	13	3.43E-07	Cellular component
GO:1904813	ficolin-1-rich granule lumen	11	4.42E-07	Cellular component
GO:0016605	PML body	7	2.34E-06	Cellular component
GO:0045121	membrane raft	6	1.19E-05	Cellular component
GO:0005125	cytokine activity	16	8.93E-14	Molecular function
GO:0061629	RNA polymerase II-specific DNA-binding transcription factor binding	17	2.94E-12	Molecular function
GO:0005126	cytokine receptor binding	15	1.10E-11	Molecular function
GO:0140297	DNA-binding transcription factor binding	18	4.05E-11	Molecular function
GO:0048018	receptor ligand activity	18	6.73E-11	Molecular function
GO:0030546	signaling receptor activator activity	18	8.48E-11	Molecular function
GO:0004879	nuclear receptor activity	8	2.51E-10	Molecular function
GO:0098531	ligand-activated transcription factor activity	8	2.51E-10	Molecular function
GO:0019902	phosphatase binding	10	7.14E-08	Molecular function
GO:0001221	transcription coregulator binding	8	9.94E-08	Molecular function

**Table 3:** KEGG analysis (top 15 signaling pathways with the smallest *p*-values)

ID	Term	Count	*P* Value
hsa05418	Fluid shear stress and atherosclerosis	30	1.85E-31
hsa04933	AGE-RAGE signaling pathway in diabetic complications	27	3.72E-31
hsa05417	Lipid and atherosclerosis	31	8.25E-27
hsa05161	Hepatitis B	22	2.16E-18
hsa05212	Pancreatic cancer	17	3.40E-18
hsa04657	IL-17 signaling pathway	18	6.37E-18
hsa05167	Kaposi sarcoma-associated herpesvirus infection	23	7.40E-18
hsa05145	Toxoplasmosis	19	7.82E-18
hsa05163	Human cytomegalovirus infection	24	1.54E-17
hsa05142	Chagas disease	18	2.99E-17
hsa04668	TNF signaling pathway	18	2.38E-16
hsa05215	Prostate cancer	17	2.79E-16
hsa04931	Insulin resistance	17	1.85E-15
hsa05140	Leishmaniasis	15	3.45E-15
hsa05205	Proteoglycans in cancer	21	4.95E-15
hsa01522	Endocrine resistance	16	7.36E-15
hsa05225	Hepatocellular carcinoma	19	1.88E-14
hsa04659	Th17 cell differentiation	16	3.62E-14
hsa04066	HIF-1 signaling pathway	16	4.20E-14
hsa04932	Non-alcoholic fatty liver disease	18	6.21E-14

**Fig. 6:**
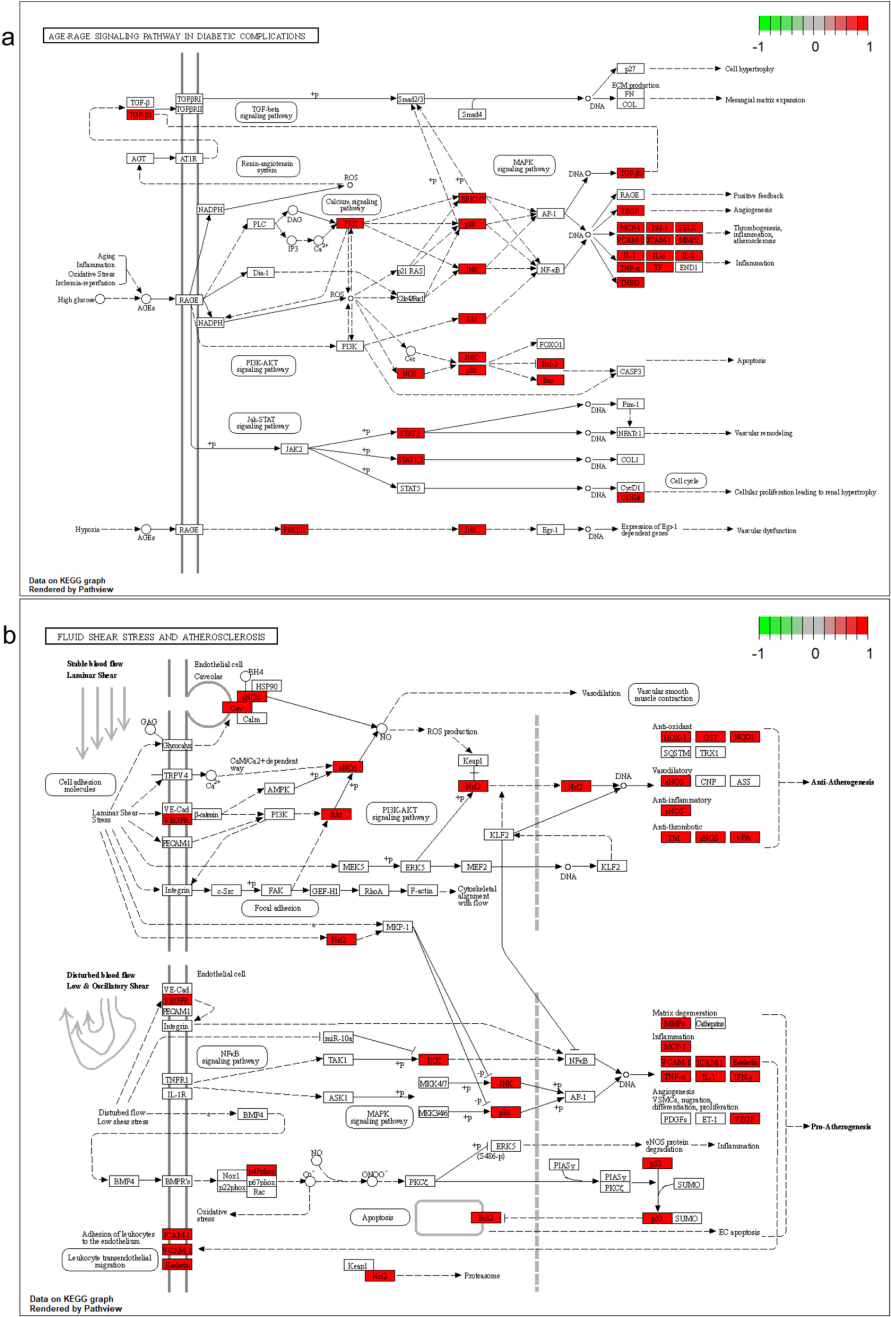
Distribution of intersecting genes in related pathways. **a** AGE-RAGE signaling pathway in diabetic complications, (**b**) Fluid shear stress and atherosclerosis signaling pathway. Red rectangles represent key targets

We found that through AGE-RAGE signaling pathway in diabetic complications and Fluid shear stress and atherosclerosis, signaling pathways were highly expressed in KEGG enrichment analysis and MCODE analysis, using the “pathview” package for the visualization of the pathways (Fig. 6). From the pathway map, we can find that PI3K/AKT signaling pathway and MAPK signaling pathway form the AGE-RAGE signaling pathway in diabetic complications and Fluid shear stress and atherosclerosis, so we think PI3K/AKT signaling pathway and MAPK signaling pathway may have a strong correlation in these pathways. But the exact mechanism may need further validation.

### Molecular docking

**Fig. 7:**
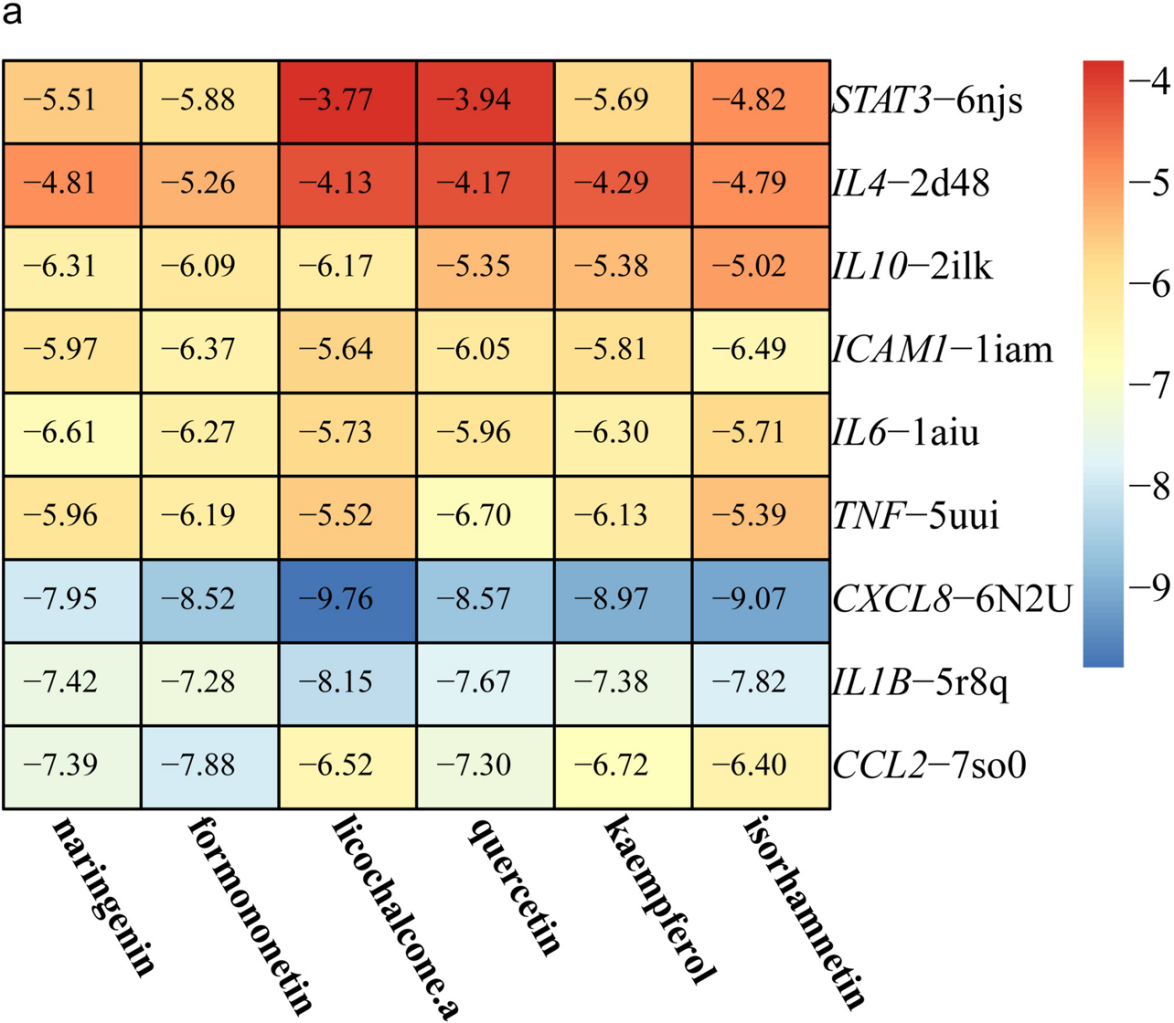
The molecular docking results of the first 6 potential compounds with 9 hub genes are scored in a heat map (unit: kcal/mol), with red representing high docking activation energy and blue representing low docking activation energy

**Fig. 8:**
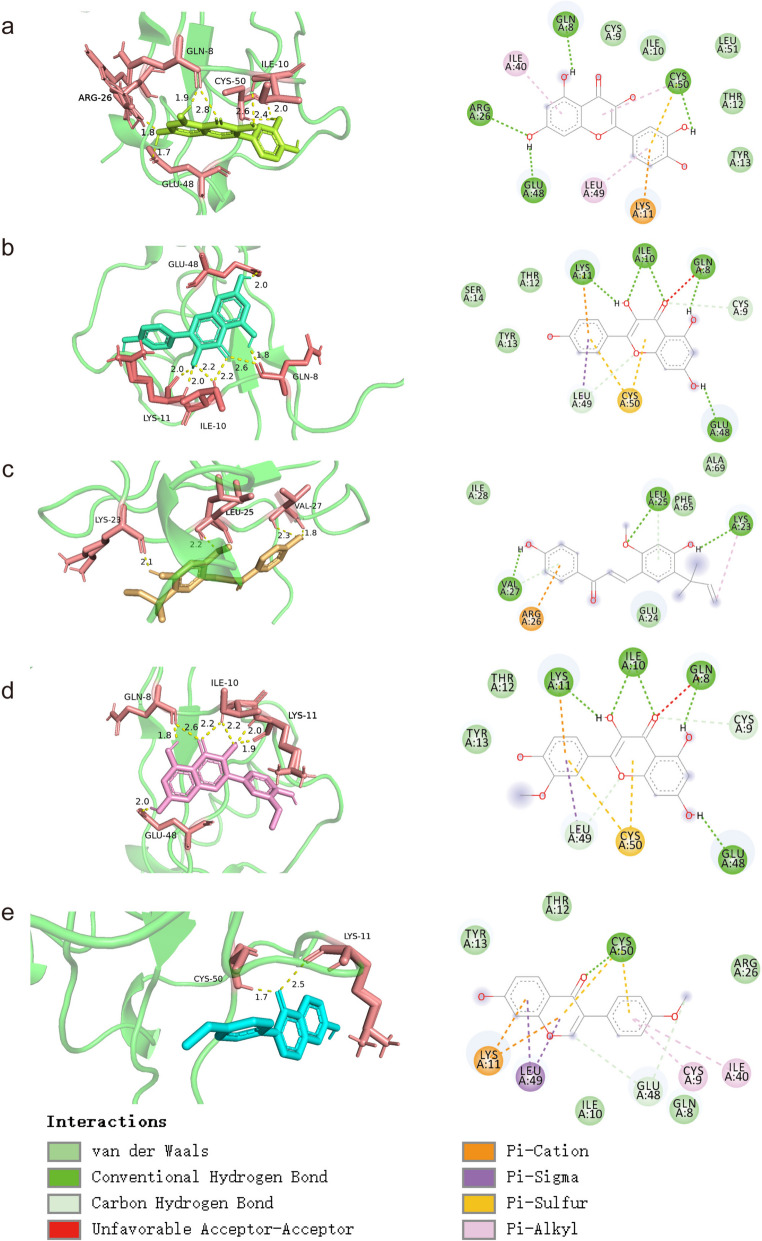
Visualization of docking results of hub gene with potential compounds, licochalcone a (**a**), isorhamnetin (**b**), kaempferol (**c**), quercetin (**d**), and formononetin (**e**) generated with *CXCL8* in 3D and 2D, with 3D results on the left and 2D results on the right

Molecular docking is a bioinformatics tool that is the process of finding the best combination of small molecules (ligands) and biomacromolecules (receptors) by intermolecular geometric matching and energy calculation of their patterns and effectiveness. Molecular docking of the first 6 active compounds and 9 hub genes was performed using autodocktools software, and a heat map of the docking results was created using “pheatmeap” (Fig. 7). Then we used PYMOL to visualize the molecular docking results in 3D and discovery studio software to visualize the docking results in 2D and selected the five compounds with the best docking activation performance to the hub gene (Fig. 8). The results showed that *CXCL8* had high binding activity to most of the compounds, with licochalcone a, isorhamnetin, kaempferol, quercetin, and formononetin having the strongest docking activity, all with docking activation energies between − 8.5 and − 9.8, which also implies stable binding. From the 2D results, the binding affinity was mainly attributed to hydrogen bonding, Pi-alkyl, pi-cation, and van der Waals forces. We found that these potential compounds have strong binding affinity to the core gene and molecular docking can only predict the binding pattern between the core gene and the potential compound. It should be noted that we still need further experimental validation of these potential compounds and targets is needed.

### Molecular dynamics simulation

Molecular dynamics can be good for discovering the relationship between proteins and ligands. We performed molecular dynamics simulations for *CXCL8*- licochalcone a, *CXCL8*- isorhamnetin and *CXCL8*- kaempferol for 100ns to evaluate the intermolecular motions, trajectories, structures, binding potentials, and conformational changes by analyzing the molecular docking results.

**Fig. 9:**
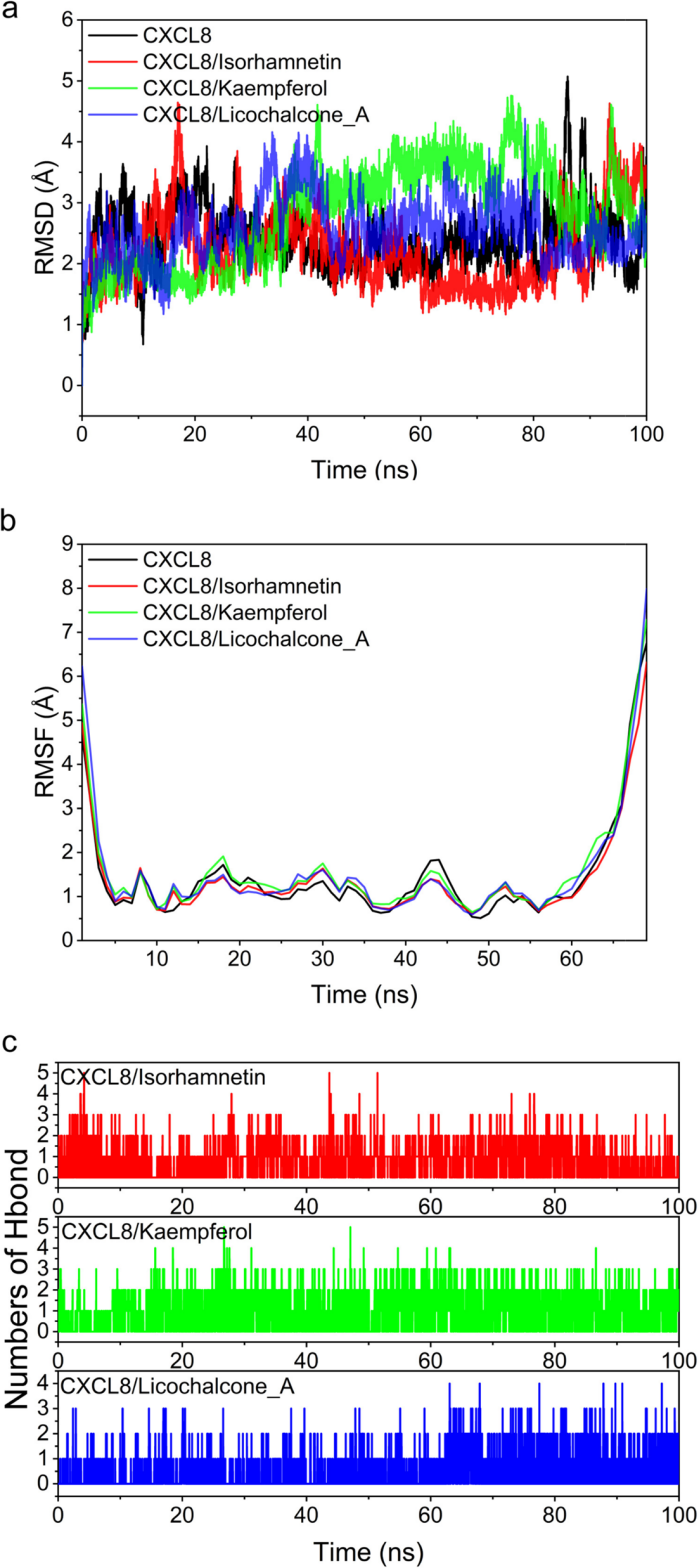
The molecular dynamics (MD) simulation of the *CXCL8*- licochalcone a complex, the *CXCL8*- isorhamnetin complex, and the *CXCL8*- kaempferol complex. **a** The RMSD plot of the complexes. **b** The RMSF plot of the complexes. **c** The number of hydrogen bonds in the complexes

Root-mean-square deviation (RMSD) can reflect the motion process of the complexes, with larger RMSD as well as more intense fluctuations indicating violent motion and, conversely, smooth motion. In conclusion, our study shows that the system maintains a stable RMSD fluctuation under the binding of three small molecules, which means that the small molecules are stable (Fig. 9a). Root Mean Square Fluctuation (RMSF) can respond to the flexibility of the protein during molecular dynamics simulations. about the same, indicating that Isorhamnetin, Kaempferol, and Licochalcone_A binding is stable and does not affect the flexibility of the protein (Fig. 9b). The hydrogen bond is one of the strongest non-covalent binding interactions, and the higher number indicates better binding. how the three groups of *CXCL8*/Isorhamnetin, *CXCL8*/Kaempferol, and *CXCL8*/Licochalcone_A generally had between 1 and 2 hydrogen bonds during the simulation (Fig. 9c). It indicates that hydrogen bonding plays a role in the binding of small molecules and proteins during kinetic simulation.

Based on the trajectories of molecular dynamics simulations, we calculated the binding energy using the MM-GBSA method, which can more accurately reflect the binding effect of small molecules and target proteins. the binding energies of *CXCL8*/Isorhamnetin, *CXCL8*/Kaempferol, and *CXCL8*/Licochalcone_A were − 15.83 ± Negative values indicate that the molecule has a binding affinity to the target protein, and lower values indicate stronger binding. Our calculations indicate that *CXCL8*/Isorhamnetin, *CXCL8*/Kaempferol, and *CXCL8*/Licochalcone_A have binding potential. By energy decomposition, we can see that the main contribution to the binding of licochalcone a, isorhamnetin, and kaempferol to *CXCL8* can be van der Waals energy, followed by electrostatic energy and non-polar solvation free energy (Table 4). In conclusion, these results demonstrate the reliability of our molecular docking results and the binding stability of *CXCL8* with licochalcone a, isorhamnetin, and kaempferol.

**Table 4:** Binding free energies and energy components predicted by MM/GBSA (kcal/mol)

System name	*CXCL8*/Isorhamnetin	*CXCL8*/Kaempferol	*CXCL8*/Licochalcone_A
**Δ*E***_**vdw**_	-21.06 ±2.10	-19.95 ±1.58	-23.53±2.29
**Δ*E***_**elec**_	-11.99 ±1.14	-10.70 ±1.66	-2.77 ±2.08
**ΔG**_**GB**_	20.56±1.91	19.15±2.53	12.68±1.61
**ΔG**_**SA**_	-3.33±0.23	-2.98±0.44	-3.30±0.31
**ΔG**_**bind**_	-15.83±2.62	-14.49±2.62	-16.93±2.84

ΔE_vdW_: van der Waals energy

ΔE_elec_: electrostatic energy

ΔG_GB_: electrostatic contribution to solvation

ΔG_SA_: non-polar contribution to solvation

ΔG_bind_: binding free energy

### Gene set enrichment analysis

**Fig. 10:**
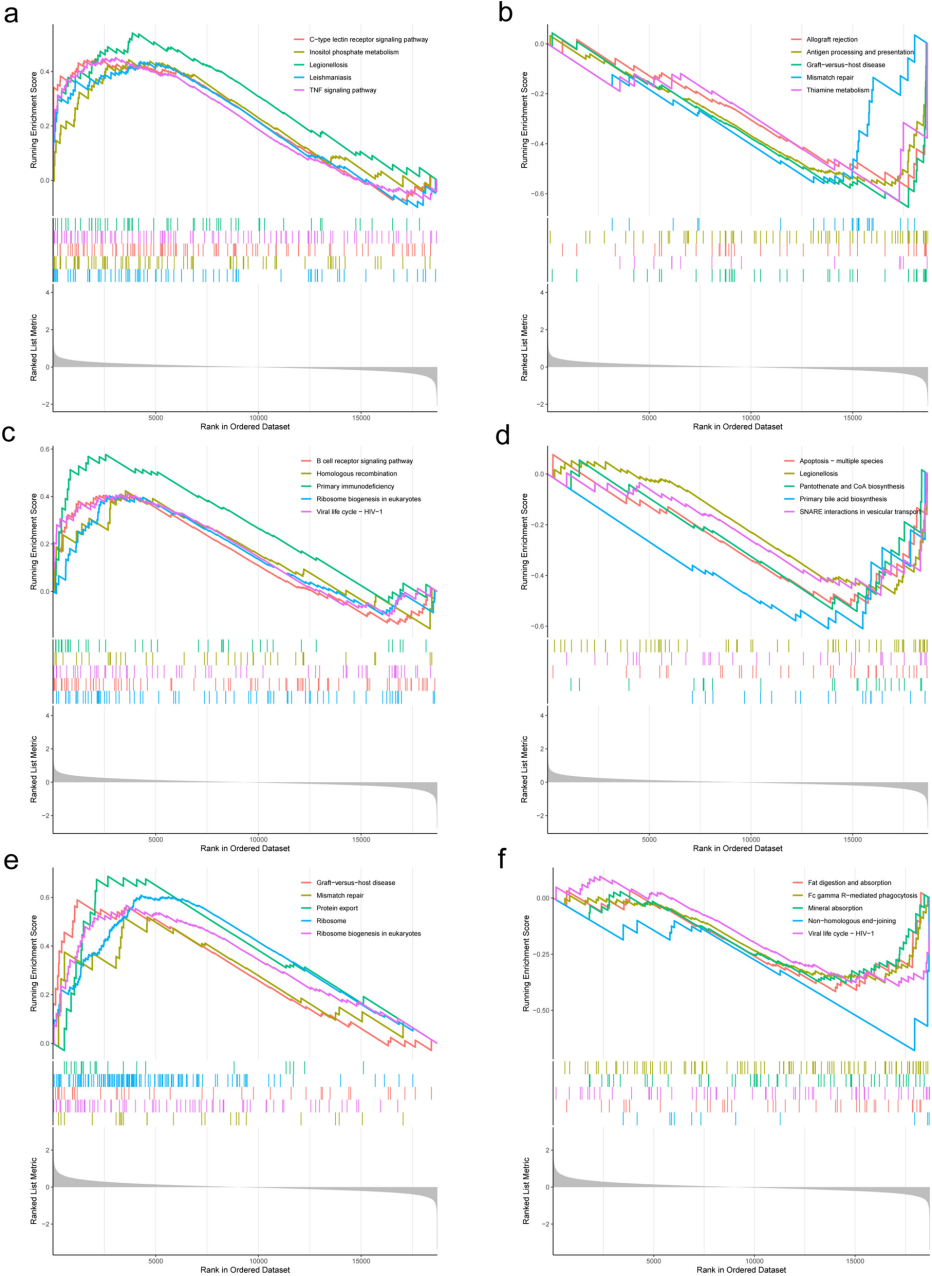
Enrichment of hub gene in GSE15932 by GSEA. **a **and **b** *CXCL8*, up (**a**), down (**b**). **c **and(d)*CCL2*, up (**c**), down (**d**). **e **and **f** *IL1B*, up (**e**), down (**f**)

GSEA enrichment analysis can analyze the regulation of genes in the dataset. Molecular docking identified genes that bind strongly to core compounds, such as *CXCL8, IL1B*, and *CCL2*. we then subjected these genes to GSEA analysis in GSE15932 to find their effects in the gene set. the GSEA results showed that *CXCL8, IL1B* and *CCL2* affect Graft-versus-host disease, Legionellosis, Primary immunodeficiency, Primary bile acid biosynthesis, Protein export and Non-homologous end-joining signaling pathways (Fig. 10). These results suggest a regulatory role of these genes in diabetes, mainly related to immunity and inflammation.

## Discussion

Diabetes not only increases the risk of cerebrovascular disease and stroke but also exacerbates neurodegenerative diseases, particularly AD [45]. Traditional Chinese medicine has gained international recognition over the course of thousands of years, with numerous prescriptions being utilized to treat a wide range of clinical conditions. In Traditional Chinese Medicine, SZRD is a classical prescription known for its sedative and tranquilizing properties, primarily used in the treatment of conditions such as “liver and blood deficiency, heat deficiency, and internal disorders" [46]. Diabetes is categorized as “depression” and “thirst” in Traditional Chinese Medicine and is believed to be primarily related to yin and qi deficiency in the patient’s body, as well as emotional and willpower disorders. Therefore, clinical treatment should focus on relieving liver depression, nourishing yin and generating fluid and tonifying qi and blood. Recent studies have shown that SZRD improves cognitive function in APP/PS1 mice and reduces levels of IL-6, IL-1β, and TNF-α [9]. These findings indicate that SZRD may have therapeutic effects on Alzheimer’s with diabetes, although its mechanism of action remains unclear. Hence, we employed a combined approach of bioinformatics, network pharmacology, and molecular docking to identify the active compounds and potential targets of SZRD in Alzheimer’s with diabetes.

We employed the principles of oral bioavailability (OB) and drug-likeness (DL) for active compound screening, and integrated them with disease targets to identify 97 intersecting targets using the D-C-T-D approach. Among these, quercetin, kaempferol, licochalcone a, isorhamnetin, formononetin, and naringenin were identified as the main core compounds, which have also been demonstrated to exhibit therapeutic effects in both diabetes and AD. Licochalcone a, a naturally occurring specific inhibitor of JNK (c-Jun N-terminal kinase), has been demonstrated to exhibit therapeutic effects in diabetic nephropathy [47]. Isorhamnetin, a regulator of the insulin signaling pathway, has been shown to improve diabetes by mitigating insulin resistance [48]. Formononetin, a naturally occurring isoflavone, acts as a non-classical agonist of PPARγ (peroxisome proliferator-activated receptor gamma), which not only treats Alzheimer’s with diabetes but also enhances fat thermogenesis to reduce obesity [49]. Naringenin has also been demonstrated to possess potent neuroprotective and antidiabetic effects [50]. Furthermore, these compounds highlight the therapeutic potential of SZRD in addressing cognitive impairment associated with diabetes.

We retrieved 1977 DEGs for diabetes and 622 DEGs for AD from the GEO database. After intersecting the targets of complementary targets and drugs, a total of 97 targets were identified. Further, we performed MCC analysis on the PPI network to identify Hub targets including *IL6, TNF, IL1B, CXCL8, IL10, CCL2, ICAM1, STAT3*, and *IL4*. Low-grade inflammation, which plays a role in the development and progression of multiple diseases, has been associated with *IL6, IL-1B, CXCL8, IL4, TNF*, and *IL10* in various diseases [51, 52]. *CCL2* acts as a ligand for the C-C chemokine receptor CCR2, which is stimulated by inflammation [53]. Meta-analysis demonstrated that *ICAM1*, a cell adhesion molecule, is elevated in the circulatory system of diabetic patients, and its levels are dose-dependently associated with the risk of diabetes [54]. *STAT3* has been proposed as a potential link between inflammation and chronic disease [55]. We observed a strong correlation between these hub genes and inflammation, which is a critical pathological feature of Alzheimer’s with diabetes. These findings imply that SZRD may primarily exert an anti-inflammatory role in treating Alzheimer’s with diabetes at the genetic level.

GO enrichment analysis revealed that SZRD regulates Alzheimer’s with diabetes through biological processes such as response to nutrient levels, oxidative stress, and cellular response to chemical stress. Oxidative stress and inflammation play a role in the development of Alzheimer’s with diabetes. Our KEGG enrichment analysis indicated that these targets were mainly enriched in pathways such as Fluid shear stress and atherosclerosis, AGE-RAGE signaling pathway in diabetic complications, Lipid and atherosclerosis, IL-17 signaling pathway, and TNF signaling pathway. Previous studies have demonstrated that Fluid shear stress and atherosclerosis, as well as the AGE-RAGE signaling pathway in diabetic complications, play a regulatory role in diabetes [56] and are also closely related to inflammation and oxidation [57]. The KEGG network and target analysis revealed the presence of inflammatory factors and oxidative stress-related proteins involved in network regulation, along with the identification of PI3K/AKT and MAPK signaling pathways in the pathway map that could potentially modulate these factors and proteins. The PI3K/Akt signaling pathway is implicated in the modulation of cytokines and plays a crucial role in regulating insulin and cognitive function in AD through the reduction of phosphorylation levels of PI3K, AKT, and mTOR [58]. Previous studies have demonstrated the involvement of PI3K in the regulation of diabetic complications. Furthermore, PI3K/AKT has been shown to play a significant role in diabetic cognitive impairment [59]. mitogen-activated protein kinase (MAPK) is a serine/threonine protein kinase that regulates multiple inflammatory responses [60], including NLRP3 and NF-κB pathways. Studies have shown that RAGE can activate the P38 MAPK/NF-κB signaling pathway, which is implicated in diabetic cognitive dysfunction [61]. Therefore, PI3K/AKT and MAPK may serve as crucial regulators in the pathways of fluid shear stress, atherosclerosis, and the AGE-RAGE signaling pathway in diabetic complications, potentially contributing to SZRD-mediated Alzheimer’s with diabetes.

Molecular docking was employed to evaluate the activity of active compounds (quercetin, kaempferol, licochalcone a, isorhamnetin, formononetin, and naringenin) with hub genes (*IL6, TNF, IL1B, CXCL8, IL10, CCL2, ICAM1, STAT3*, and *IL4*). The binding affinities were predominantly <-4.25 kcal/mol, indicating robust interactions between most compounds and hub genes, with kaempferol, licochalcone a, and isorhamnetin exhibiting the strongest docking activities with hub gene *CXCL8*. Subsequently, we conducted molecular dynamics simulations to further investigate this, and the results further confirmed the strong binding stability of kaempferol, licochalcone a, and isorhamnetin with hub gene *CXCL8*. Lastly, we performed GSEA analysis on *CXCL8*, *IL1B*, and *CCL2*, three hub genes with significant docking activity, using the results of molecular docking. The GSEA analysis further demonstrated the pivotal roles of *CXCL8*, *IL1B*, and *CCL2* in diabetes through immune and inflammatory mechanisms. In conclusion, our study elucidates the potential role of active compounds and hub genes of SZRD in the treatment of Alzheimer’s with diabetes.

## Limitation

However, there are some limitations of the study in this paper. First, from the perspective of Traditional Chinese Medicine, suanzaoren is considered the core component of this formula. Most of the predicted compounds identified in our study belong to gancao, rather than suanzaoren, which raises concerns about the reliability of the prediction. Secondly, the active compounds of SZRD remain unidentified and could be compensated by additional research methods such as LC/MS, metabolomics, and pharmacokinetics. Finally, validation through animal and cellular experiments was not conducted due to constraints such as time limitations, which could be considered in future research. Our experiments mainly illustrate the preliminary therapeutic effect of SZRD on Alzheimer’s with diabetes, while our results predict the key role played by these active ingredients.

## Conclusion

In summary, this study applied bioinformatics, network pharmacology, molecular docking, and molecular dynamics simulation to predict the pharmacological effects and molecular mechanisms of SZRD in Alzheimer’s with diabetes. Licochalcone A, isorhamnetin, and kaempferol have the potential to serve as the primary active ingredients in SZRD treatment. The results of molecular dynamics simulations also suggest a strong association between the hub gene and Licochalcone A, isorhamnetin, and kaempferol. Our results provide a theoretical basis for subsequent experimental verification.

## Data Availability

Data will be made available on request.

## Abbreviations

TCM: Traditional Chinese Medicine
SZRD: Suanzaoren Decoction
AD: Alzheimer's disease
DEG: Differentially expressed genes
GSEA: Gene Set Enrichment Analysis
Suanzaoren, SZR: Ziziphus jujuba Mill
Gancao, GC: Glycyrrhiza uralensis Fisch
Zhimu, ZM: Anemarrhena asphodeloides Bunge
Fuling, FL: Poria cocos (Schw.) Wolf
Chuanxiong, CX: Ligusticum acuminatum Franch
GEO: Gene Expression Omnibus
BC: Betweenness centrality
CC: Closeness centrality
ASPL: Average shortest path length
MCC: Maximum population centrality
D-C-T-D: Drug-compound-target-disease
PPI: Protein-protein interaction
BP: Biological process
CC: Cellular component
MF: Molecular function
HF: Hartree-Fock
OB: Oral bioavailability
DL: Drug similarity
PME: Particle mesh Ewald
ES: Enrichment scores
